# Advances in hormonal therapies for hormone naïve and castration-resistant prostate cancers with or without previous chemotherapy

**DOI:** 10.1186/s40164-016-0046-1

**Published:** 2016-06-22

**Authors:** Thy Pham, Martin C. Sadowski, Huika Li, Derek J. Richard, Michael C. d’Emden, Kerry Richard

**Affiliations:** 1 Conjoint Endocrine Laboratory, Chemical Pathology, Pathology Queensland, Queensland Health, Level 9, Bancroft Centre, 300 Herston Road, Herston, QLD 4029 Australia; 2 Australian Prostate Cancer Research Centre–Queensland, Institute of Health and Biomedical Innovation, Queensland University of Technology, Princess Alexandra Hospital, Translational Research Institute, Brisbane, QLD 4102 Australia; 3 School of Biomedical Sciences, Queensland University of Technology, Brisbane, QLD 4000 Australia; 4 Department of Endocrinology and Diabetes, Royal Brisbane and Women’s Hospital, Herston, QLD 4029 Australia

**Keywords:** Prostate cancer, Testosterone, Androgen receptor, Androgen deprivation therapy, Hormone naïve, Castration-resistant

## Abstract

Hormonal manipulation plays a significant role in the treatment of advanced hormone naïve prostate cancer and castration-resistant prostate cancer (CRPC) with or without previous chemotherapy. Combination of gonadotropin releasing hormone (GnRH) agonists and androgen receptor (AR) antagonists (combined androgen blockade; CAB) is the first line therapy for advanced hormone naïve prostate cancer, but current strategies are developing novel GnRH antagonists to overcome disadvantages associated with GnRH agonist monotherapy and CAB in the clinical setting. Abiraterone acetate and enzalutamide are hormonal agents currently available for patients with CRPC and are both shown to improve overall survival versus placebo. Recently, in clinical trials, testosterone has been administered in cycles with existing surgical and chemical androgen deprivation therapies (ADT) (intermittent therapy) to CRPC patients of different stages (low risk, metastatic) to abate symptoms of testosterone deficiency and reduce cost of treatment from current hormonal therapies for patients with CRPC. This review will provide an overview on the therapeutic roles of hormonal manipulation in advanced hormone naïve and castration-resistant prostate cancers, as well as the development of novel hormonal therapies currently in preclinical and clinical trials.

## Background

Prostate cancer is the most reported male cancer as well as the second leading cause of cancer-related deaths in Western men, excluding non-melanoma skin diseases [1]. Ever since 1941, when it was discovered that lowering testosterone levels via orchiectomy or estrogen injections improved symptoms in patients with metastatic, exogenous hormone naïve disease, androgen deprivation therapy (ADT) became the mainstay treatment for locally advanced prostate cancer (clinical tumor stages T3–T4, PSA > 20 ng/mL) [2]. However, despite reducing testosterone production to castrate levels (≤50 ng/dL), many patients will relapse and develop castration-resistant disease within 2–3 years post treatment, that is often more aggressive, currently incurable and has a poor prognosis with only 16–18 months of survival [3, 4]. This review will provide an overview on the therapeutic roles of hormonal manipulation in advanced hormone naïve and castration-resistant prostate cancers, as well as the development of novel hormonal therapies currently in preclinical and clinical trials. This will include the potential use of testosterone, although critically involved in cancer growth and progression, as a therapy for patients with castration-resistant prostate cancer (CRPC).

### Physiology of androgen biosynthesis and action

Testosterone is a hormone produced primarily by Leydig cells of testes and in smaller quantities by the adrenal glands [5, 6]. The secretion of testosterone in testes and adrenal glands is regulated by luteinising hormone (LH) and adrenocorticotropic hormone (ACTH), respectively; both of which are derived from the pituitary gland [5–8]. Inactive androgen receptors (AR) are located in the cytoplasm and are bound by heat-shock protein (HSP) chaperones including HSP90, HSP70 and HSP40 [9]. Following activation by androgens, AR dissociates from the HSP complex, dimerizes and binds onto HSP27 which allows localization of AR to the nucleus and subsequent transcription of downstream target genes such as prostate-specific antigen (PSA), a glycoprotein that is also referred as kallikrein-3 (KLK3) [9–11]. Regulation of AR-specific genes is important for the development and maturation of prostate and other male sexual characteristics. Studies have shown that testosterone deficiency is associated with erectile dysfunction, low libido, reduced muscle and physical strength [12, 13]. Testosterone is further required for the growth and survival of prostate tumors, and ADT is commonly used to suppress disease progression among patients [2, 14, 15].

Interestingly, low testosterone levels have been linked to increased risk of developing high-grade prostate cancer [16, 17]. A study by Schatzl et al. [17] found that prostate cancer patients with partial androgen deficiency (n = 52; total serum testosterone <3.0 ng/mL) at diagnosis had significantly higher Gleason scores than patients with normal serum testosterone levels (n = 104; >3.0 ng/mL) (Gleason score 7.4 ± 1.2 vs. 6.2 ± 1.4, respectively; p = 0.001). Other studies have reported an association between low testosterone, prostate cancer risk and tumor reoccurrence [18, 19], suggesting that ADT may have adverse effects on patients with prostate cancer. Nevertheless ADT has been shown to significantly improve clinical outcomes in combination with other therapies (e.g. radiotherapy) which are discussed in further detail in this review.

### Locally advanced hormone naïve prostate cancer

#### GnRH agonists—overview

Prior to the use of immunotherapy and chemotherapy, ADT is the first-line therapy for locally advanced or high-risk hormone naïve prostate cancer (clinical tumor stages T3–T4, PSA > 20 ng/mL) [20]. Gonadotropin releasing hormone (GnRH) agonists are the standard hormonal agents for ADT due to their reversibility, allowing intermittent use when required. GnRH agonists can also prevent complications associated with orchiectomy, such as physical discomfort [20, 21]. However recently, a 10-year study by Van Hemelrijck et al. has suggested that patients treated with GnRH agonists were at higher risk for deep-venous thrombosis (DVT) and pulmonary embolism than those who underwent orchiectomy (Absolute risk value 4.08 vs. 1.40, respectively) [22].

GnRH agonists are very long acting drugs which bind to the gonadotrophin receptors on the pituitary gland. Their initial binding causes a marked increase in luteinising hormones (LH) and follicle stimulating hormones (FSH) secretion, resulting in a transient increase in testosterone levels [23, 24]. This “flare” can induce adverse symptoms such as bone pain, spinal cord compression, cardiovascular events, and ureteral blockage among patients [24, 25]. Thus, to abate this effect, GnRH agonists are usually accompanied with AR antagonists and this is often referred as combined androgen blockade (CAB) therapy (Table 1). Within approximately 3–4 weeks, the pituitary GnRH receptors undergo desensitization and suppression, thus lowering serum testosterone to castrate levels (≤50 ng/dL) [26]. There are currently four United States (US) Food and Drug Administration (FDA) -approved GnRH agonists for advanced hormone naïve prostate cancer patients: goserelin acetate (Zoladex^®^), leuprolide (Lupron Depot^®^), histrelin acetate (Supprelin LA^®^) and triptorelin pamaote (Trelstar Depot^®^, Trelstar LA^®^) (Table 1) [27–31].

**Table 1:** FDA-approved hormonal therapies for advanced hormone naïve prostate cancer and metastasis castration-resistant prostate cancer

Stage of prostate cancer	Hormonal agent	Function	Year of FDA approval	Reference
Advanced, hormone naïve	Goserelin acetate	GnRH agonist	1989	[27, 28]
	Leuprolide acetate	GnRH agonist	1989	[27, 29]
	Histrelin acetate	GnRH agonist	1991	[27, 30]
	Triptorelin pamoate	GnRH agonist	2000	[27, 31]
	Abarelix	GnRH antagonist	2003	[27, 48]
	Degarelix	GnRH antagonist	2008	[27, 49]
	Bicalutamide	AR antagonist	1995	[27, 126]
	Flutamide	AR antagonist	1989	[27, 127]
Castrate-resistant	Abiraterone acetate	CYP17 inhibitor	2011^b^	[27, 94]
			2012^a^	[96, 99]
	Enzalutamide	AR antagonist	2012^b^	[27, 101]
			2014^a^	[102, 128]

*GnRH* gonadotropin releasing hormone, *AR* androgen receptor

^a^Patients without previous treatment with docetaxel (chemotherapy naïve)

^b^Patients treated and progressed from docetaxel (post-chemotherapy)

#### GnRH agonists—clinical advancement for prostate cancer therapy

At present, anticancer strategies have taken an interest in supplementing GnRH agonists with concurrent radiotherapies to optimize efficacy of treatment for locally advanced or high-risk prostate cancer [32]. The 10-year overall survival rate for patients treated with radiotherapy and adjuvant ADT with goserelin, is evidently greater than radiation monotherapy (49–58.1 vs. 39–39.8 %, respectively; p < 0.002) [33, 34]. Likewise, a combination of ADT and radiotherapy is more effective when compared to ADT alone [35]. As shown by the SPCG-7 trial, the cumulative incidence for prostate cancer-specific mortality at 10 years was 23.9 % for the group treated with ADT alone (3.75 or 11.25 mg GnRH agonist leuprorelin plus 250 mg AR antagonist flutamide for 3 months) and 11.9 % in the group treated with ADT in combination with radiotherapy [35].

Further studies have sought to deliver goserelin to the tumor via nanoparticle carriers. In the clinical setting, nanoparticles are macromolecular, conductive materials (metal or semi-metal; size range 5–250 nm) that carry and deliver anticancer drugs to the vicinity of the tumor and solely penetrate target tissue by converting absorbed light photons at wavelengths near the infrared spectrum (800–2500 nm) into heat, and this is often referred as photothermal therapy [36–38]. The selective delivery of nanoparticles to solid tumors among patients is highly dependent on their large molecular size and how cancer cells develop in contrast to normal cells [36]. Most solid tumors increase the production of new blood vessels (angiogenesis) to enhance vascular permeability and provide more nutrients to sustain their growth [39]. However, unlike normal blood vessels, the endothelial junctions of tumor blood vessels are more loose due to poor development and lack efficient lymphatic drainage, which allows the macromolecular nanoparticles to easily pass through as well as accumulate at the tumor site for effective treatment [39]. This phenomenon known as the “enhanced permeability and retention” (EPR) effect differentiates most solid tumors from normal tissues and is essential for the therapeutic properties of nanoparticles [36, 39].

Goserelin-conjugated gold rod-shaped nanoparticles (commonly known as nanorods) were used in a recent in vivo study by Wolfe et al. and these demonstrated a significant increase in radiosensitization of PC3 xenograft models, as compared to standard pegylated gold nanorods (1.36 ± 0.06 vs. 1.19 ± 0.04, respectively). Treatment with the goserelin-conjugated gold nanorods plus radiotherapy delayed tumor growth by 17 ± 1 days versus standard pegylated gold nanorods plus radiotherapy or radiotherapy alone in PC3 xenograft models (p < 0.001) [40]. Likewise, another study reported that goserelin loaded nanoparticles can induce growth inhibition and apoptosis in LNCaP and DU145 cell lines [41]. These preclinical findings are promising and warrant further investigation.

Efficacy of GnRH agonists in prostate cancer treatment is likely due to their specificity toward GnRH receptors found in the pituitary gland; however increasing evidence has indicated that the presence of GnRH receptors in tumors of the prostate, as demonstrated by the in vitro and in vivo studies mentioned previously, and of other organs, such as breast, uterus and ovary might be their additional molecular targets [40–42]. There are currently two types of GnRH receptors (GnRH receptors I and II) found in prostate cancer cells in vitro [43, 44].

Better outcomes with radiotherapy are achieved with long-term adjuvant ADT (2–3 years) than short-term adjuvant ADT (<2 years). A phase III randomised, controlled trial (ClinicalTrials.gov, number NCT02175212) suggests that advanced or high-risk prostate cancer patients receiving long-term adjuvant ADT (3.6 mg subcutaneous goserelin; after 1 month, 10.8 mg was given every 3 months for 28 months) plus high dose radiotherapy (dose range 76–82 Gy) within 5 years had significantly improved biochemical disease-free survival (90 vs. 81 %, respectively; p = 0.01), metastasis-free survival (94 vs. 83 %, respectively; p = 0.009), and overall survival (95 vs. 86 %, respectively; p = 0.01) versus those in the short-term ADT plus high dose radiotherapy group (same analog and dose regimen, for 4 months) [45].

As with radiotherapy, chemotherapeutic drugs such as docetaxel and estramustine are far more beneficial with adjuvant goserelin ADT. A randomised phase III trial (ClinicalTrials.gov, number NCT00055731) with 413 advanced or high-risk prostate cancer patients were administered to either ADT (10.8 mg goserelin every 3 months for 36 months) with docetaxel (70 mg/m^2^; four cycles on Day 2) and estramustine (10 mg/kg per day; for 5 days every 3 weeks) or ADT alone [46]. In median follow-up of 8.8 years, patients who received ADT, docetaxel and estramustine had fewer relapses and mortality than those who received only ADT; 88 of 207 (43 %) patients versus 111 of 206 (54 %) patients, respectively [46]. 8-year relapse-free survival was 62 % in the ADT, docetaxel and estramustine group compared to 50 % in the ADT only group whilst reporting no treatment-related deaths (p = 0.017) [46].

Clinical observations have shown no significant differences in efficacy between the two GnRH agonists, leuprolide (3.75 and 7.5 mg) and goserelin (3.6 mg) [47]. It is noteworthy, however, that a substantial proportion of patients (26.3, 25 and 35 % of patients who received 3.75 mg leuprolide, 7.5 mg leuprolide, and 3.6 mg goserelin, respectively) did not achieve castration levels of testosterone (≤50 ng/dL) regardless of which GnRH agonist was used in the study [47]. Future research to improve the efficacy of chemical castration may therefore be needed.

#### GnRH antagonists—overview

There are two second-line GnRH antagonists for patients with locally advanced or high-risk prostate cancer, degarelix (Firmagon^®^) and abarelix (Plenaxis^®^), although the latter has been restricted to patients with no alternative therapy because of associated severe allergic reactions (Table 1) [48, 49], where 3 of the 81 (3.7 %) patients had immediate-onset histamine surges and systemic allergic reactions upon treatment [24, 50].

GnRH antagonists directly inhibit pituitary GnRH receptors and do not cause an initial LH, FSH and testosterone surge or “flare” as opposed to GnRH agonists, thereby suppressing testosterone to castrate levels (≤50 ng/dL) with minimal delay. A randomized, controlled phase III trial (CS21) has shown that patients receiving either low or high dose of the GnRH antagonist degarelix (Firmagon^®^) subcutaneously for locally advanced or high-risk prostate cancer (240 mg followed by either 80 or 160 mg monthly, respectively) were more likely to reach castration levels of testosterone by day 3 post treatment than those receiving a GnRH agonist (leuprolide; intramuscular; 7.5 mg monthly), with 95.5–96.1 % patients vs. 0 % patients, respectively [51]. Combined with this, PSA was decreased significantly faster in the degarelix group versus the leuprolide group [51].

#### GnRH antagonists—clinical advancement for prostate cancer therapy

To date, there are clear advantages of GnRH antagonists (particularly degarelix) over GnRH agonists [51–56]. At 1 year follow-up, degarelix (subcutaneous; 240 mg followed by 80 mg every month) has reduced recurrence of elevated PSA in locally advanced or high-risk patients compared to those receiving leuprolide (7.5 mg every month) (7.7 vs. 12.9 %, respectively; baseline PSA > 20 ng/mL; p = 0.04) [52]. Likewise, a study by Albertson et al. revealed a significant reduction in cardiovascular risk after 1 year treatment with degarelix when compared to GnRH agonists (p = 0.002) [53]. Klotz et al. [54] reported that patients treated with degarelix had significant improvements in PSA progression-free survival (baseline PSA > 20 ng/mL; p = 0.052), overall survival (p = 0.023) and fewer musculoskeletal events and urinary tract events compared to the leuprolide group and the goserelin group.

GnRH agonists and GnRH antagonists have differential effects on FSH secretion. A study by Crawford et al. [57] reported a significant suppression in FSH levels from baseline among patients who switched from GnRH agonist leuprolide to GnRH antagonist degarelix for 1 year. Median FSH was 1.20 IU/L in the degarelix group (subcutaneous; 240 mg followed by 80 mg every month) and 4.40 IU/L the leuprolide group (intramuscular; 7.5 mg) (p < 0.0001) [57]. Elevated FSH levels were common among patients who received treatment with either a GnRH agonist or orchiectomy, or those who have switched from degarelix to leuprolide [58, 59]. In vitro studies detected overexpression of FSH receptors in prostate cancer compared to benign prostatic epithelium [60]. Moreover, a FSH value more than the lowest tertile (>4.8 mIU/mL) may be associated with shorter time to CRPC progression [61]. Taken together, the efficacy of GnRH antagonists may be due to lack of FSH stimulation as opposed to GnRH agonists.

Because degarelix is the only efficient GnRH antagonist for prostate cancer, alternative GnRH antagonists are currently in development [62–66]. A 10-amino acid peptide (decapeptide) GnRH antagonist acyline is reported to significantly lower serum LH, FSH and testosterone levels below baseline values for 15 and 21 days among healthy men (cohort 1 and cohort 2) following treatment with acyline as either a single dose (subcutaneous; 300 µg/kg on day 0; cohort 1 baseline values: LH = 3.3 ± 0.7 IU/L, FSH = 2.7 ± 0.8 IU/L, testosterone = 21.6 ± 4.2 nmol/L) or multiple doses (subcutaneous; 75 µg/kg on days 0, 2, 4, 6 and 8; cohort 2 baseline values: LH = 4.0 ± 0.4 IU/L, FSH = 2.0 ± 0.3 IU/L, testosterone = 26.1 ± 4.2 nmol/L), respectively (p < 0.05) [65]. In a recent study by Festuccia et al. ozarelix, a decapeptide GnRH antagonist, significantly restores cell sensitivity to human recombinant tumor necrosis factor-related apoptosis inducing ligand (TRAIL)-mediated apoptosis in androgen-independent prostate cancer cell lines DU145 and PC3 (~60–70 % apoptotic cells; 20 ng/mL ozarelix plus 500 ng/mL TRAIL) versus cells treated with TRAIL alone (~10 % apoptotic cells; 500 ng/mL TRAIL) [66]. TRAIL is a member of the tumor necrosis factor (TNF) superfamily and resistance is usually acquired by cancer tissue to prevent apoptosis and subsequent cell death [67]. A novel non-peptide GnRH antagonist TAK-375 (Relugolix^®^) has recently completed phase I trials with healthy men and is progressing onto phase II [62]. Unlike peptide GnRH antagonist degarelix, non-peptide GnRH antagonists can be taken orally which can prevent disadvantages associated with subcutaneous and intramuscular routes of administration; for instance, adverse reactions to injection site and high dosage intake for efficacy [68].

GnRH antagonists are proven to be effective therapies clinically. However, the currently inevitable disease progression to castration-resistant prostate cancer (CRPC) renders this therapy, like all other therapies for advanced prostate cancer, inefficient. In a novel study, patients with locally advanced or high-risk prostate cancer underwent radical prostatectomy (RP) with or without prior neoadjuvant degarelix (subcutaneous 240 mg; administered 7 days before study) [69]. RP samples were collected from each patient for analysis. Degarelix-treated samples had lower mRNA expression of cell proliferation gene *Ki*-*67* (*MKI67*) and cell cycle regulatory gene *cyclin D1* (*CCND1*), as opposed to untreated samples (p = 0.003) [69]. Expression levels of AR-regulated genes *PSA* (*KLK3*) and fatty acid synthase (*FASN*) were significantly downregulated following degarelix treatment (p = 0.005 and p = 0.0002, respectively). However, malignant epithelial cells in degarelix-treated samples had higher *estrogen receptor 1* (*ESR1*) expression versus untreated samples (24 vs. 8 %, respectively; p = 0.0002) [69]. This therefore indicates that GnRH antagonists may not inhibit intrinsic pathways mediated from growth promoters such as ESR1 which could assist in the development of castration resistance and further disease progression among patients.

### Castration-resistant prostate cancer (CRPC)

#### Overview

Many studies have reported that CRPC remains androgen-dependent and is driven by intra-tumoral androgens [70–73]. This phenomenon is due to AR reactivation which most commonly occurs through either overexpression or mutation of wild-type AR, with about 30 and 20–40 % present in CRPC patients, respectively [74–76]. Overexpression of wild-type AR may be induced by the long noncoding HOX transcript antisense RNA (*HOTAIR*) which has been shown to prevent binding of AR protein to the E3 ubiquitin ligase MDM2 which directs AR’s proteasomal degradation [77]. AR overexpression often leads to enhanced hypersensitivity toward low circulating androgens, which enables prostate cancer cells to further progress despite the use of ADT [78, 79]. Chen et al. [78] found that AR shRNA-infected prostate cancer tumors in castrated male mice grew more slowly compared to the empty vector control. This indicates a distinct selection for cells that avoided AR knockdown for tumor growth. Consistent with this finding, in vivo and in vitro studies have shown that AR upregulates M-phase cell cycle regulatory genes such as *CDC20, CDK1* and *UBE2C* and stimulate proliferation [80]. These observations suggest that increased AR regulation is important for developing resistance to hormonal therapy.

Mutations in the ligand-binding domain (LBD) of AR are known to switch non-steroidal AR antagonists into agonists; a phenomenon known as anti-androgen withdrawal syndrome (AAWS). Recent studies indicate that AAWS occurs with AR antagonists enzalutamide (MDV3100) and ARN-509 due to an AR-F876L point mutation [81, 82]. Interestingly, exposure to CAB therapy (GnRH agonist plus AR antagonist) has minimal effect on the secretion of testosterone precursor androstenediol and is shown to stimulate antagonist-to-agonist switch T877 mutation in AR [83]. A clinical study by Taplin et al. reported an increase in the AR-T877A point mutation among 5 of 16 (31.3 %) patients administrated with CAB therapy (ADT via LnRH agonist or orchiectomy plus AR antagonist flutamide) versus 0 of 17 (0 %) patients administered to ADT only [84]. Although CAB therapy can avoid testosterone surges when compared to ADT monotherapy, its use remains controversial due to increased risk of AAWS and resistance to castration.

Other mechanisms of CRPC involve AR splice variants (AR-Vs) which lack the C-terminus including the LBD and are constitutively active in the absence of androgens [85]. Detection of AR splice variants 6 (AR-V6; contiguously-spliced AR exons 1/2/3/2b) and 7 (AR-V7 contiguously-spliced AR exons 1/2/3/CE3) in CRPC tumors is associated with resistance to enzalutamide, an AR antagonist that specifically targets the LBD of AR [86, 87]. Recently, third generation AR antagonists were developed that are directed against domains other than the LBD of AR to improve efficacy of CRPC treatment. EPI-001 is a novel AR antagonist that targets the N-terminal domain of AR [88]. Consisting of four stereoisomers (EPI-002, EPI-003, EPI-004 and EPI-005), EPI-001 is revealed to inhibit transcription of AR-regulated genes in response to synthetic androgen R1881 [88]. EPI-506, a derivative of EPI-001, also targets the N-terminal domain of AR and is currently in phase I trials (ClinicalTrials.gov, number NCT02606123) [89]. The DNA-binding domain (DBD) is an alternative site for inhibition of AR-Vs. Recent evidences have shown that DBD-targeting compound VPC-14449 (4-(4-(4,5-bromo-1H-imidazol-1-yl)thiazol-2-yl)morpholine) (100 mg/kg; 4 days) suppressed tumor size and serum PSA levels in LNCaP xenograft models [90]. Existing antimicrobial drugs can induce suppression of full length AR (AR with C-terminal of LBD) and AR splice variant expression including nigericin (antibiotic) and niclosamide (anthelmintics) [91, 92].

#### Abiraterone acetate

Abiraterone acetate (Zytiga^®^) is an inhibitor of CYP17 (17α-hydroxy/17,20-lyase), an enzyme responsible for the intratumoral androgen synthesis of adrenal androgens and testosterone precursor dehydroepiandrosterone (DHEA) [93]. Clinically, abiraterone acetate (oral; four 250 mg tablets once daily with oral prednisone 5 mg twice daily) is shown to significantly improve median overall survival compared to placebo in metastatic CRPC (mCRPC; histologically confirmed prostate cancer, N1/M1, more than two [or three in other studies] consecutive rises in PSA levels over 25 % above nadir value following surgical or chemical ADT for at least 1 week) patients with or without previous chemotherapy [94–97]. A randomized phase III trial demonstrates that abiraterone acetate significantly reduced pain intensity in chemotherapy naïve mCRPC patients versus the placebo group (26.7 vs. 18.4 months, respectively; p = 0.0490) [98]. Abiraterone acetate received FDA approval for mCRPC patients with or without previous chemotherapy in 2011 and 2012, respectively [27, 94, 96, 99] (Table 1).

Since its approval, the treatment of mCRPC patients with abiraterone acetate has become popular. Truven Health Analytics MarketScan^®^ and electronic medical record (EMR) databases in the US have shown that 67 % mCRPC patients had abiraterone acetate as first-line therapy in 2013 compared to only 15 % using chemotherapeutic drug docetaxel [100]. In 2010, 1 year before the approval of abiraterone acetate, about 91 % mCRPC patients received docetaxel as first-line therapy [100]. The positive clinical outcomes associated with abiraterone acetate highlights the important role of androgen biosynthesis in CRPC.

#### Enzalutamide

Enzalutamide (Xtandi^®^) is an AR antagonist that has been approved by FDA for post-chemotherapeutic and chemotherapy naïve mCRPC patients in 2012 and 2014, respectively (Table 1) [101, 102]. A double-blind, phase II study, AFFIRM, has shown that enzalutamide (oral; four 40 mg capsules for median 8.3 months) improved median overall survival compared to the placebo group with previous chemotherapy (18.4 vs. 13.6 months, respectively; p < 0.001) [101]. A similar trend occurred for mCRPC patients without previous chemotherapy in another study, PREVAIL (32.4 vs. 30.2 months, respectively) [102].

There is debate on whether enzalutamide is more effective among patients with advanced hormone naïve prostate cancer than standard ADT (GnRH agonist plus AR antagonist), with a novel randomized phase II trial currently being performed (ClinicalTrials.gov, number NCT02278185) [103].

#### Potential use of testosterone for CRPC therapy

For decades it was thought that testosterone played a pivotal role in prostate cancer onset; however, recent studies have found no statistically significant association between testosterone administration (testosterone replacement therapy) and prostate cancer risk or mortality [104–107]. A recent study has shown that testosterone might have therapeutic benefits for patients with asymptomatic mCRPC (histologically confirmed prostate cancer, rising PSA levels following surgical or chemical ADT for at least 4 weeks, castrate serum testosterone ≤50 ng/dL, no cancer-related pain, ≤5 asymptomatic bone metastases, ≤10 total sites of metastases including soft tissue) [108]. In 6 of the 14 (42.9 %) patients there were PSA reductions after three 28-day cycles of testosterone (intramuscular; 400 mg) plus 14 days of the chemotherapy drug etoposide (oral; 100 mg) [108]. Of these 14 patients, 4 (28.6 %) patients had >50 % PSA reductions from mean baseline (21.7 ng/mL; range 1.4–819.1 ng/mL). Low-grade (grade 1–2) alopecia (56.3 %), fatigue (56.3 %) and nausea (62.5 %) were most common among patients post treatment, followed by lower incidences of high-grade (grade 3–4) neutropenia (12.5 %) and pulmonary embolism (12.5 %) [108]. Likewise, a prior study by Morris et al. reported low-grade fatigue as the most common toxicity among all three cohorts of patients (75 %; n = 3 [cohort 1], n = 3 [cohort 2] and n = 6 [cohort 3]) with progressive mCRPC (histologically confirmed prostate cancer, 25 % increase in PSA over three tests) following high-dose exogenous testosterone (transdermal; 5 mg; patch [cohorts 1 and 2] or gel [cohort 3]; 1 week, 4 weeks, or until progression for cohorts 1, 2 and 3, respectively) [109]. No high-grade toxicities related to treatment were observed [109]. PSA suppression was seen in 1 of 3 (33.3 %), 2 of 3 (66.7 %) and 4 of 6 (66.7 %) patients from cohorts 1, 2 and 3, respectively [109]. However, despite this unique finding, the timing in which testosterone supplementation occurs is critical. Patients must previously be treated with ADT via surgical castration or GnRH agonist for at least 1 year prior to study to avoid potential adverse effects associated with significantly higher levels of testosterone or flares among patients with existing bone metastases and nodal metastases causing increased rates of fractures at metastatic site and ureteral obstruction, respectively [108, 109]. Higher levels of testosterone may also lead to increased risk of cancer progression. A number of publications have documented the potential for testosterone therapy to induce progression among patients with hormone naïve prostate cancer [2, 15]. On the contrary, a randomized phase I study reported no significant relationship between dose or serum testosterone levels and median time to progression (p = 0.072 and p = 0.14, respectively) among patients with low risk CRPC (histologically confirmed prostate cancer, PSA ≤ 3.0 ng/mL; rising PSA after surgical or chemical ADT) [110]. The recent studies highlight that testosterone is well tolerated among patients with asymptomatic and progressive mCRPC, and low risk CRPC but future investigation is needed [108–110].

In patients with increasing PSA levels due to relapse following local therapy (radical prostatectomy, radiation therapy) treatment with testosterone decreased PSA to undetectable levels (<0.05 ng/ml). Feltquate et al. have shown that 2 of 9 (22.2 %) patients with non-castrate testosterone levels (mean 382 ng/dL; range 181–654 ng/dL) and PSA relapse after radiation therapy achieved undetectable PSA levels after 28-day cycles of testosterone repletion (transdermal gel; 5 g daily) on days 1 to 7 plus GnRH agonist (either leuprolide 7.5 mg intramuscular or goserelin 3.6 mg subcutaneous) on day 1 [111]. With the same dose and regimen, 8 of 17 (47 %) patients with PSA relapse following radical prostatectomy had achieved undetectable PSA levels [111]. However, prior to testosterone repletion, patients were required to undergo 12-week induction cycles of GnRH agonist (leuprolide 7.5 mg intramuscular or goserelin 3.6 mg subcutaneous) on day 1 plus AR antagonist bicalutamide (50 mg daily) on days 1 to 28 to deplete PSA levels to <1 ng/mL [111]. Patients who failed to achieve a PSA of <1 ng/mL after the initial induction did not received testosterone repletion in the study [111]. Likewise, a phase II trial reported that 5 of 38 (13 %) non-castrate patients (testosterone levels >150 ng/dL) with histologically confirmed prostate cancer and PSA relapse following prostatectomy or radiation therapy achieved “treatment-specific” undetectable PSA endpoint (PSA < 0.05 ng/mL or PSA < 0.5 ng/mL for patients with prior prostatectomy or radiation therapy, respectively) at 18 months after receiving nine 3-week cycles of testosterone repletion (transdermal gel; 5 g daily for 3 days) in combination with 3-month depot leuprolide (intramuscular; 22.5 mg) on Day 1 of cycles 1, 5 and 9, and docetaxel (70 mg/m^2^) on Day 1 per cycle [112]. Of these 5 patients, 3 had prostatectomy and 2 had radiation therapy prior to study [112].

As demonstrated by recent clinical trials, testosterone administration is generally supplemented with cycles of ADT to deplete serum androgen and PSA levels for a certain period of time. The process of cycling between androgen repletion and depletion, known as intermittent therapy, was first introduced in 1986 by Klotz et al. [113] to minimize toxicity and adverse side effects associated with continuous ADT. However, in addition to stopping the use of ADT, recent studies have taken interest in modifying androgen repletion with testosterone therapy to not only alleviate side effects associated with ADT but also to potentially reduce cost of hormonal treatment for CRPC patients (Table 2) [114–123]. Interestingly, intermittent therapy is thought to prolong androgen dependence in prostate cancer when compared to continuous ADT; however, at present there is no statistically significant evidence to suggest that intermittent therapy without testosterone administration during the repletion stage improves overall survival of patients with PSA relapse following local therapy versus continuous ADT [124, 125]. Long term randomised clinical trials should be performed to determine if testosterone administration during intermittent therapy can significantly affect overall survival among patients with prostate cancer.

**Table 2:** Prices of several hormonal agents for prostate cancer in Australia (as of January 2016)

Hormonal agent/s	Form and strength	Price ($AUD)		Reference
		Full price	PBS (maximum number of repeats for PBS price)	
Advanced or high-risk hormone naïve prostate cancer (clinical tumor stages T3–T4, PSA > 20 ng/mL)				
Goserelin acetate	3.6 mg implant	$323.50	$38.30 (5)	[114]
	10.8 g implant	$1101.16	$38.30 (1)	[114]
Triptorelin acetate	3.75 mg injection	$413.75	$38.30 (5)	[115]
	22.5 mg injection	$2123.34	$38.30 (0)	[115]
Goserelin acetate and Bicalutamide	3.6 mg implant and 50 mg tablet (28 tablets), respectively	$472.81	$38.30 (5)	[116]
	10.8 mg implant and 50 mg tablet (28 tablets), respectively	$1240.02	$38.30 (0)	[116]
Leuprorelin (or leuprolide) acetate	30 mg injection	$1442.09	$38.30 (1)	[117]
Degarelix	80 mg injection	$413.75	$38.30 (5)	[118]
	120 mg injection	$432.92	$38.30 (0)	[118]
Bicalutamide	50 mg tablet (28 tablets)	$98.40	$38.30 (5)	[119]
Flutamide	250 mg tablets (100 tablets)	$169.70	$38.30 (5)	[120]
Nilutamide	150 mg tablet (30 tablets)	$223.68	$38.30 (5)	[121]
Metastatic castration-resistant prostate cancer (mCRPC) (histologically confirmed prostate cancer, N1/M1, more than two [or three in other studies] consecutive rises in PSA levels over 25 % above nadir value following surgical or chemical ADT for at least 1 week)				
Abiraterone acetate	250 mg tablet (120 tablets)	$3600.41	$38.30 (2)	[122]
Enzalutamide	40 mg capsule (112 capsules)	$3700.17	$38.30 (2)	[123]

*PBS* pharmaceutical benefits scheme

## Conclusions

Hormonal therapy is the mainstay of treatment in the clinical management of pre- and post-castration-resistant prostate cancers. Until recently, GnRH agonists comprised the first-line therapy for advanced hormone naïve prostate cancer and are generally combined with AR antagonists to avoid initial testosterone flares, and either radiotherapy or chemotherapy to improve clinical outcomes. Progress has been made since the discovery of degarelix with the development of novel GnRH antagonists such as the non-peptide GnRH antagonist TAK-375 which is entering phase II trials. Due to advanced knowledge on AR reactivation and castration resistance, two hormonal agents have been approved by FDA for patients with CRPC: abiraterone acetate and enzalutamide. Abiraterone acetate has particularly gained vast interest because it directly inhibits biosynthesis of testosterone precursors, unlike current hormonal therapies. Although still early in clinical trials, the use of testosterone therapy could be expanded to patients with CRPC. It would be interesting to see if testosterone therapy could be translated into larger randomized clinical trials for further analysis, and subsequently allow the development of more effective therapeutic methods to manage the disease.

## Abbreviations

ACTH: adrenocorticotropic hormones
ADT: androgen deprivation therapies
AR: androgen receptor
CRPC: castration-resistant prostate cancer
CAB: combined androgen blockade
DHEA: dehydroepiandrosterone
DBD: DNA-binding domain
FSH: follicle stimulating hormones
GnRH: gonadotropin releasing hormone
HSP: heat-shock protein
KLK3: kallikrein-3
LH: luteinising hormones
PSA: prostate-specific antigen
